# A Comparison of Electrochemical Performance of Carbon Aerogels with Adsorption Metal Ions for Super Capacitors

**DOI:** 10.3390/ma11112271

**Published:** 2018-11-14

**Authors:** Xiaoxi Dong, Yuelong Xu, Shasha Wang, Junping Zhao, Bin Ren, Lihui Zhang, Zhenfa Liu

**Affiliations:** 1 School of Chemical Engineering and Technology, Hebei University of Technology, Tianjin 300000, China; a13292005986@126.com (X.D.); wssnys@126.com (S.W.); 2 Institute of Energy Resources, Hebei Academy of Sciences, Shijiazhuang 050081, China; xudalong.cool@163.com (Y.X.); zjp39698@126.com (J.Z.); renbints@163.com (B.R.); zlhkxy@126.com (L.Z.); 3 Hebei Engineering Research Center for Water Saving in Industry, Shijiazhuang 050081, China

**Keywords:** carbon aerogels, adsorption behavior, metal ions, super capacitor, electrochemical performance

## Abstract

Environmental problems caused by metal ions have caused widespread concern in recent years. In this work, carbon aerogels (CAs) adsorbing different metal ions were prepared. The adsorption performance and kinetics of metal ions (Cu(II), Cr(VI), and Fe(III)) on carbon aerogels were systematically investigated. The results indicated that the maximum adsorption capacity of Cu(II) was 424 mg·g^−1^ in 600 mg·L^−1^ copper solution. Adsorption performances of Cu(II), Cr(VI), and Fe(III) on CAs well fitted with a pseudo-second-order kinetic model. The structures and morphologies of metal-containing samples were characterized by scanning electron micrographs (SEM), Energy Dispersive Spectrometer (EDS), transmission electron microscope (TEM), and X-ray diffraction (XRD). The results demonstrated that the texture and electrochemical performance of CAs adsorbing metal ions exhibited a clear change. The specific surface area of CAs for adsorbing copper ions was 450 m^2^·g^−1^ and they showed a small average pore diameter (7.16 nm). Furthermore, CAs adsorbing metals could be used for the super capacitor. The specific capacitance of CAs adsorbing copper ions could reach 255 F·g^−1^ at a current density of 1.0 A·g^−1^. The CA-Cu electrode materials exhibited excellent reversibility with a cycling efficiency of 97% after 5000 cycles.

## 1. Introduction

Water pollution and soil contamination from heavy metal ions, which resulted from various industrial manufacturing, had been a worldwide environmental problem. Therefore, the removal of heavy metal ions from wastewater and soil have attracted more and more attention in recent years. Traditional methods for removing heavy metal ions included adsorption, precipitation, flocculation, reverse osmosis, ion exchange, and electrochemical treatments [1,2,3,4,5]. Recently, adsorption has become an effective, economic, and easily operational method to control metal pollutants. Common adsorbents included carbon nanotubes, activated carbon, and carbon aerogels.

Pekala et al. [6] first discovered carbon aerogels in 1989. Carbon aerogels (CAs) had nanostructures, a high specific surface area, porosities, and high electric conductivities. These excellent characteristics made CAs broadly applicable in environmental treatments and some good results have been achieved by using these materials [7,8]. Kadirvelu et al. [9] studied CAs as adsorbents that adsorb metal ions in multi-component systems. The results revealed that they were competitive for the adsorptions of the three metal ions. The adsorption of each ion conformed to the Langmuir and Freundlich model. Kabbashi et al. [10] found that carbon nanotubes could adsorb lead ions to remove them from aqueous solutions. The results showed that the maximum adsorption capacity was 102.04 mg·g^−1^ and the removal rate was 96.03%. Li et al. [11] found the maximum adsorption capacity to be 55.25 mg·g^−1^ for copper ions by CAs in aqueous solutions in the presence of a surfactant. Wang et al. [12] found that, due to the oxygen-containing functional groups of the acidified carbon nanotubes that were treated by concentrated nitric acid, the nanotubes could mainly adsorb Pb(II) through the formation of chemical complexes. The oxygen-containing functional groups accounted for 75.3% of all the Pb(II) adsorption capacity. These examples clearly demonstrated that carbon materials were widely used in wastewater treatment due to their low production cost and ability to remove a significant fraction of the heavy metal ions. However, some adsorbent adsorbing heavy metal ions had to be thrown away and this led to secondary pollution due to a lack of a proper treatment method. In this work, the CAs could be recycled and used for super capacitors after adsorbing heavy metals.

The super capacitors have been widely used and studied because of their advantages such as long service life and high power density [13,14]. CAs as electrode materials could be used for super capacitors due to a high specific surface area and electric conductivity. After the CAs adsorbed the metal ions, we could use the modified materials for super capacitors. Wang et al. [15] discovered that hybrid electrode material of carbon fiber and vanadium nitride (CF@VN) was prepared by adsorbing scrap metal ions at 800 °C under a mixed atmosphere of NH_3_ and N_2_. The results showed that the specific capacitance of the CF@VN material was 104.05 F·g^−1^ at the current density of 0.5 A·g^−1^. Hao et al. [16] used chitosan coated oxygen-containing functional carbon nanotubes to remove toxic metal ions and carbonized the mixing materials. Electrochemical measurements showed that the specific capacitance of Cu-loaded and Cr N-loaded carbon composites was 144.9 and 114.9 F·g^−1^ at 2 mV·s^−1,^ respectively. Yu et al. [17] reduced graphene oxide-coated cellulose fiber (rGO@CF) as adsorbent in which methylene blue (MB), alizarin red S (ARS), and indigo carmine (IC) were quickly and easily adsorbed and removed from the aqueous solution and then loaded on rGO@CF paper. The flexible supercapacitors/rGO@CF electrode exhibited excellent electrochemical performances.

In this case, carbon aerogels were used to adsorb Cu(II), Cr(VI), and Fe(III). The adsorption isotherms were suitable for the Langmuir model. In addition, metal-adsorbed carbon aerogels could be used as super capacitor materials. By observing electrochemical measurements, the specific capacitance of carbon aerogels adsorbing copper ions could reach 255 F·g^−1^ at 1.0 A·g^−1^. Carbon aerogels as adsorbent provided a new way to remove metal ions. The corresponding materials exhibited an excellent electrochemical performance and could be used as super capacitor electrode materials and resource utilization.

## 2. Materials and Methods

### 2.1. Preparation of the Carbon Aerogels

Phloroglucinol (P), resorcinol (R), and formaldehyde (F) were used as raw materials to prepare the carbon aerogels. The molar ratio of P/R was 0.14. In addition, sodium carbonate (C) and ascorbic acid (Vc) were used as catalysts. The ratios of C/(P + R) and Vc/(P + R) were 0.002 and 0.02, respectively. The mass concentration of the distilled water (P + R + F + C + Vc) in the solution was 45%. All of the solutions of raw materials were prepared in distilled water and stored in glass bottles. Afterward, the glass bottles were placed into an ultrasonic bath to obtain uniform solutions. The bottles were left in the water bath at 60 °C for 2 days, which generated gels. The wet gels were placed in acetone for 3 days and the solvent was replaced every day. The gel was placed in a tube furnace, evacuated, and then purged with nitrogen. Afterward, the gels were carbonized by a temperature programming method. The temperature of the carbonation process was set so that uniform holes would form. Lastly, the CAs were obtained.

#### 2.1.2. Determination of the Metal Ions

Fe(II) was determined by spectrophotometry. A total of 4.21 g (NH_4_)_2_Fe(SO_4_)·6H_2_O was dissolved in distilled water to prepare a standard iron solution. The standard iron solution (50 mL, 600 mg·L^−1^) and 5 mg of CAs were added to a beaker and stirred on a magnetic stirrer for 1 h, 2 h, 3 h, 5 h, 7 h, 9 h, 11 h, and 18 h. The stirred solution was centrifuged, 20 mL of the supernatant was transferred to a 100 mL volumetric flask, and then it was diluted with water to the mark. The absorbance was measured at its maximum absorption wavelength (230 nm) and the concentration of Fe(II) versus absorbance was plotted [18]. Distilled water was used as a reference during the entire operation. The method for measuring the concentration of Cr (VI) (2.27 g K_2_CrO_4_) was almost the same as that of Fe(III). However, Cr (VI) was measured at a maximum absorption wavelength of 370 nm [19]. When measuring the concentration of Cu(II) (3.39 g Cu(NO_3_)_2_·3H_2_O), the copper reagent, which served as the color developing agent was added, and the pH was adjusted to 9 with ammonia (13 mol/L). The concentration was determined at the maximum absorption wavelength (452 nm) [20].

#### 2.1.3. Preparation of Metallic CA Electrodes

The CA powder (3 g) was mixed with 200 mL of iron solution, chromium solution, and copper solution (600 mg·L^−1^, respectively) and the mixtures were stirred for 24 h so that the metal ions were fully adsorbed and then the samples were dried in an oven at 45 °C. Lastly, the samples were carbonized at 600 °C. The CAs alone were used as the contrast. We named the carbonized samples as CA-Fe, CA-Cr, CA-Cu, and blank, respectively, and they were denoted CA-X and the blank. Additionally, the CAs adsorbing copper ions that were not carbonized were denoted CA-Cu-0. After carbonization, all the samples were ball milled for 6 h and then passed through a 200 mesh sieve. The samples were then added to polytetrafluoroethylene (PTFE) at a mass ratio of 95:5. Approximately 40 mL of deionized water was added and the mixtures were stirred for 6 h and dried in a drying oven at 105 °C overnight. Lastly, the dried substances were placed on foam nickel. The electrodes were prepared by using a tablet press with 10 MPa. Then the samples were dried and weighed. To test the electrochemical performance, the samples were soaked in 6 M KOH electrolyte for 24 h.

### 2.2. Carbon Aerogel Adsorption Kinetics

The kinetics of the CAs adsorption processes were studied. The appropriate kinetic model was selected to fit the adsorption process. The relevant adsorption kinetic parameters were calculated, which was important for investigating the adsorption processes. In this experiment, pseudo-first-order kinetic equations and pseudo-second-order kinetic equations were used to fit the adsorption process and experimental data of the CAs with metal ions. The expression for the pseudo-first-order kinetic equation is shown below [21].

(1) $$\ln\left(Q_{e}-Q_{t}\right)\;=\;\ln Q_{e}\;-\;\frac{k_{1}}{2.303}t$$

The expression for the pseudo-second-order kinetic equation is shown below.

(2) $$\frac{t}{Q_{t}}\;=\;\frac{1}{k_{2}Q_{e}^{2}}\;+\;\frac{t}{Q_{e}}\;$$

In the equation, *k*_1_ (min^−1^) and *k*_2_ (g·(mg·min)^−1^) stood for the rate constants of pseudo-first-order and pseudo-second-order adsorption, respectively. *t* was the reaction time (min), $Q_{t}$ was the amount of adsorption at time *t* (mg·g^−1^), and *Q_e_* was the balanced adsorption capacity (mg·g^−1^). In Equation (1), plotting $\ln\left(Q_{e}-Q_{t}\right)$ against t linearizes the data. If a linear function was obtained, it could be used as a pseudo-first-order kinetic model. Similarly, in Equation (2), $\frac{t}{Q_{t}}$ plotted against t linearizes the data. If a linear function was obtained, it could be used as a pseudo-second-order kinetic model.

In 1918, Langmuir [22] proposed the theory of monolayer adsorption. It was believed that, when the gas molecules collided with the solid surface, the adsorption rate was equivalent to the rate that it desorbed from the solid surface, which corresponds to a dynamic equilibrium process. The basic mathematical expression of the Langmuir isotherm equation is shown below.

(3) $$\frac{C_{e}}{Q_{e}}\;=\;\frac{1}{Q_{m}b}\;+\;\frac{C_{e}}{Q_{m}}\;$$

In this equation, *Q_e_* is the balanced adsorption capacity (mg·g^−1^), *Q_m_* is the monolayer saturated adsorption (mg·g^−1^), b is the adsorption equilibrium constant (L·mg^−1^), and C_e_ is the concentration of adsorbates in the system at the adsorption equilibrium (mg·L^−1^).

In addition, the adsorption capacity of carbon aerogels could be assessed based on the amount of adsorbed metal (*Q_e_*). The amount of adsorbed metal was calculated by the following formula [21].

(4) $$q_{e}\;=\;\frac{C_{0}-C_{e}}{m}V$$

In Formula (4), *q_e_* is the adsorption capacity (mg·g^−1^), *C*_0_ and *C_e_* are the concentrations of metal ions in the solution before and after adsorption, respectively (mg·L^−1^), m is the quantity of adding adsorbent (g), and V is the volume of the solution containing metal ions (L).

### 2.3. Characterization

The gels were carbonized by tube furnace (OTF-1200X-5L, HeFei Kejing Materials Technology Co. Ltd., Hefei, China) at 600 °C. The Brunaure-Emmett-Teller method and Density-Functional-Theory (DFT) model calculated the specific surface area and the micropore surface area (ASAP 2420, Norcross, GA, USA). The pore volumes and pore size were used in the Barrett-Joyner-Halenda (BJH) model. The degassing temperature was maintained at 90 °C for 1 h and then increased to 250 °C for 6 h. The carrier gas was N_2_. The surface morphologies of the samples were analyzed by SEM (s-4800-I, Tescan, Brno, Czech Republic), which were equipped with an APOLLO EDS immobilizing the sample with conduction glue. The internal morphology was observed by TEM (JEM-2100 Plus, JEOL. Co. Ltd., Tokyo, Japan), which the ground CA-X powder was placed in an absolute ethanol solution and the ultrasonic wave was evenly uniform and dropped into the micro-grid. XRD (X-ray diffraction, Cu target, λ = 1.54 Å and Kα radiation, UItima IV X-ray diffractometer, Rigaku, Tokyo, Japan) was used to identify the specific peak to metals and the peaks characteristic of the CAs. The surface compositions of CA-X were characterized by XPS (PHI5600, PHI, Lafayette, LA, USA). A UV-vis spectrophotometer (TU-1900, Beijing Persee Instruments Co. Ltd., Beijing, China) was used to measure the metal ions concentration and characterize the adsorption capacity of CAs. Electrochemical performances were measured with a GAMRY Interface 1000 electrochemical workstation. All the measurements used a three-electrode system with a saturated calomel electrode (SCE) and Pt as a reference and counter electrode. The CA-X and blank samples were the working electrode. In this work, cycle voltammetry (CV, scanning rate 0.1~20 mV·s^−1^, scanning voltage −0.8 to 0 V), electrochemical impedance spectroscopy (EIS), and charge and discharge testing (voltage range −0.8 to 0 V, current density range 0.5 to 3.0 A·g^−1^) were used for the investigation. The formula to calculate the specific capacitance was shown below [23].

(5) $$C_{m}\;=\;2I∆t/\left(m∆V\right)\;$$

In this case, *C_m_* was the single-electrode specific capacitance (F·g^−1^), I was the charge current (A), ∆t was the charge time (s), m was the quality (g), and ∆V was the potential window (V).

## 3. Results and Discussion

### 3.1. Adsorption Kinetics of CA-X

**Figure 1:**
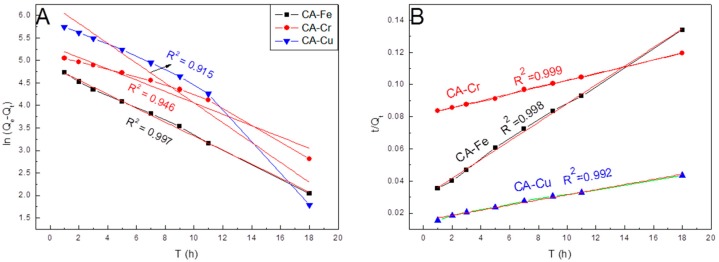
**Figure 1**. (**A**) Pseudo-first-order kinetic model and (**B**) pseudo-second-order kinetic model.

The results of the pseudo-first-order kinetic and the pseudo-second-order kinetic models under different stirring time were shown in Figure 1. From Figure 1A, in the pseudo-first-order kinetic model, the correlation coefficients (R^2^) of CA-Fe, CA-Cr, and CA-Cu were 0.997, 0.946, and 0.915, respectively. In the pseudo-second-order kinetic model (Figure 1B), the R^2^ values of CA-Fe, CA-Cr, and CA-Cu were 0.998, 0.999, and 0.992, respectively. Clearly, the pseudo-second-order kinetic model had a higher R^2^ (R^2^ > 0.99). The adsorbing metal ions followed pseudo-second-order kinetics model.

**Figure 2:**
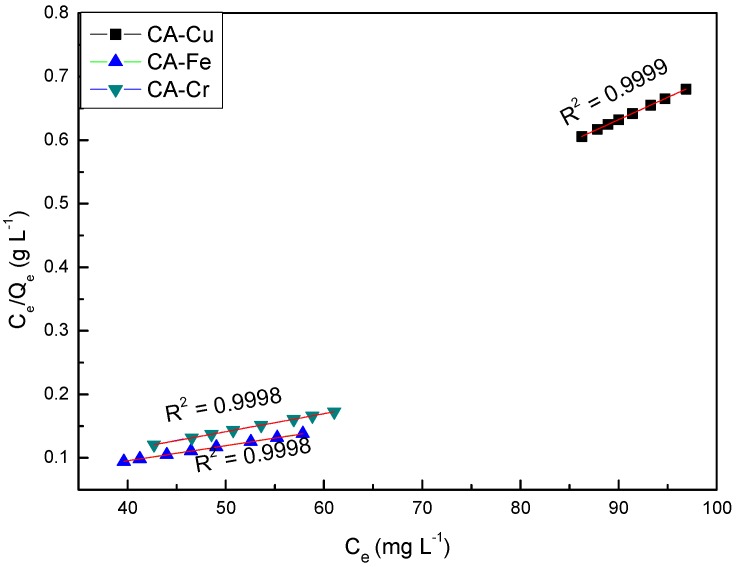
Langmuir isothermal adsorption model for CA-X.

The results of fitting the Langmuir isothermal adsorption equation to the experimental data were shown in Figure 2. According to Figure 1, CA-Cu had the largest correlation coefficient (R^2^ = 0.9999), which indicated that the adsorption process of Cu(II) on CAs was well fitted with physical adsorption. The adsorption capacities of carbon materials were largely dependent on the surface functional groups such as –COOH and –C=O, which could be introduced on the surface of the CAs. These functional groups mainly contributed to the adsorption of Cu (II) on the surface of the CAs [24]. Furthermore, the smaller ionic radius of Cu(II) was more beneficial for the adsorption on CAs [25]. Therefore, the experimental data were consistent with the Langmuir model of single-layer adsorption.

**Figure 3:**
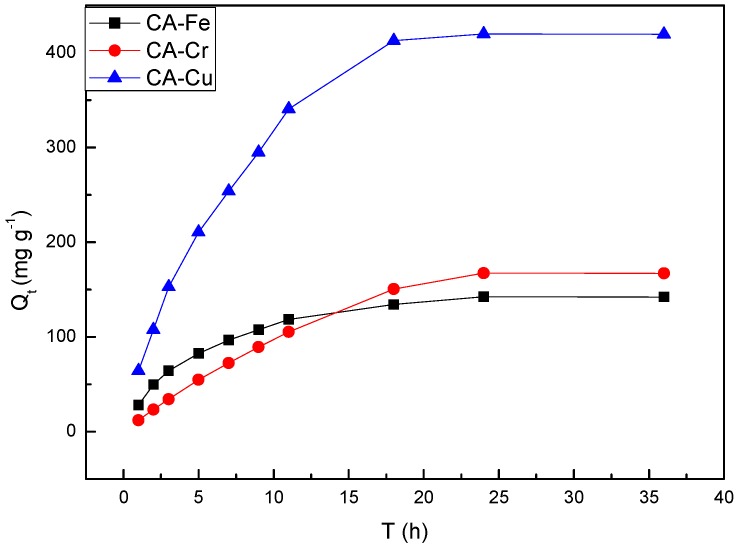
Adsorption isotherm of CA-X.

Figure 3 showed the results of adsorption of metal ions by CAs. With increasing stirring time, the amounts of adsorbed metal by the CAs increased. In particularly, the maximum amount of Cu(II) adsorbed by the CAs was 424 mg·g^−1^. As shown in Table 1, the maximum adsorption amounts of CA-Fe and CA-Cr were 133 mg·g^−1^ and 139 mg·g^−1^, respectively. The reason for this was the strong electrostatic attraction between the metal ions and the CAs [26]. Before UV measured the absorbance, the copper solution was adjusted to a pH of approximately 9 with ammonia. When the standard solution containing iron was prepared, 6 M HNO_3_ was added to dissolve the (NH_4_)_2_Fe(SO_4_)·6H_2_O and the pH was approximately 6. The pH value of the standard chromium solution was 7 by using HNO_3_ and NaOH to adjust. In acidic solutions, metal ions competed with H^+^ for the adsorption sites, which resulted in the reduction of adsorption of the heavy metal ions. Therefore, Fe(III) had the smallest adsorption capacity (133 mg·g^−1^) and the adsorption amount of Cr(VI) was 139 mg·g^−1^. However, in an alkaline solution, the proportion of (CuOH)^+^ was large, the average charge density was small, and the electronegativity was high. At the same time, more anion adsorption sites provided by functional groups such as carboxyl and carbonyl and surface complexation with Cu(II) resulted in increasing adsorption [27]. The maximum adsorption capacity of different adsorbent for the removal metal ions was shown in Table 2.

### 3.2. Pore structure of the CA-X

**Figure 4:**
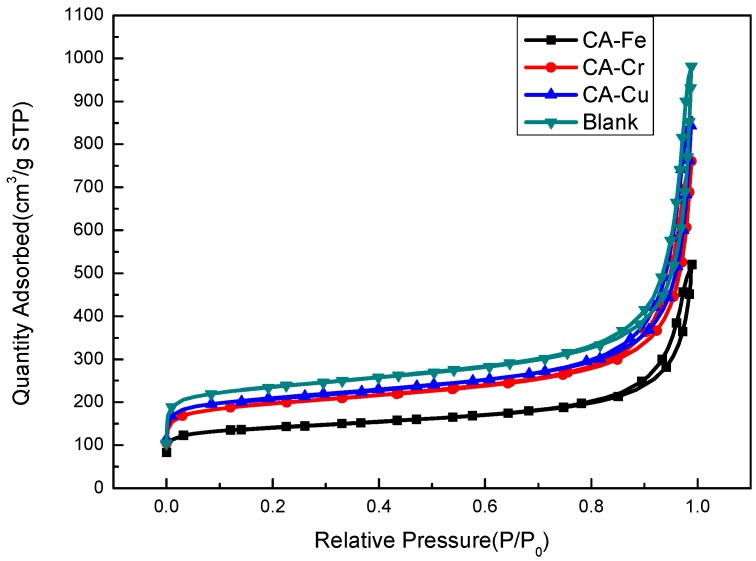
Nitrogen adsorption-desorption isotherms of the CA-X and the blank.

The N_2_ adsorption-desorption isotherms of CA-Fe, CA-Cr, CA-Cu, and the blank at 77 K were shown in Figure 4. All the adsorption-desorption isotherms of CA-X and blank were classified as type IV, according to the IUPAC [32]. At lower relative pressures, the inflection points of CA-Fe, CA-Cr, and CA-Cu were gentler, which suggests less presence of micropores in CAs, which was caused by the metal ions adsorption on CAs [33,34]. When the relative pressure P/P_0_ > 0.8, all the curves had an H1 type hysteresis loop, which indicated that there were mesopores and macropores in the materials and capillary condensation occurred.

**Figure 5:**
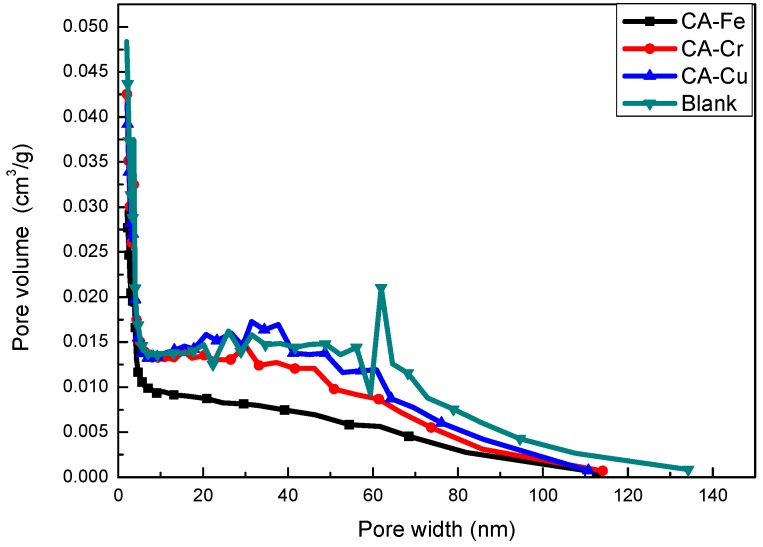
Pore size distribution in the CA-X samples and the blank.

Figure 5 showed the pore size distribution. As shown in Figure 5, all curves showed typical hierarchical porosity and the materials were composed of micropores, mesopores, and macropores, which were advantageous for adsorbing metals. Adsorption occurred mainly in micropores while adsorption in micropores required mesoporous/macroporous transitions. From Figure 4, we could understand that the hysteresis loops of the samples were consistent with the H1 model in the IUPAC [32] regulations, which indicates that the materials had good channel connectivity.

Table 3 showed the specific surface area (S_BET_), total pore volume (V_total_), micropore surface area (S_micro_), and average pore diameter (D_average_) of the CA-Fe, CA-Cr, CA-Cu, and blank samples. Clearly, the CA-Cu sample had the smallest S_BET_, V_total_, S_micro_, and D_average_ values. The result was attributed to the occupation of adsorbing metal ions on CAs. As presented in Table 3, CA-Cu exhibited a lower specific surface area than other samples, which indicated that the percentage of micropores on carbon aerogels had a significant decrease. The blank sample exhibited a noticeable large pore volume than CA-X because the metal ions adsorption on CAs contributed to the decrease of the pore volume. The analysis result was in accordance with the N_2_ adsorption-desorption isotherms discussion. At the same time, the decreasing tendency of the specific surface area showed that the metal ions adsorption process was physical adsorption.

### 3.3. SEM and TEM of CA-X

**Figure 6:**
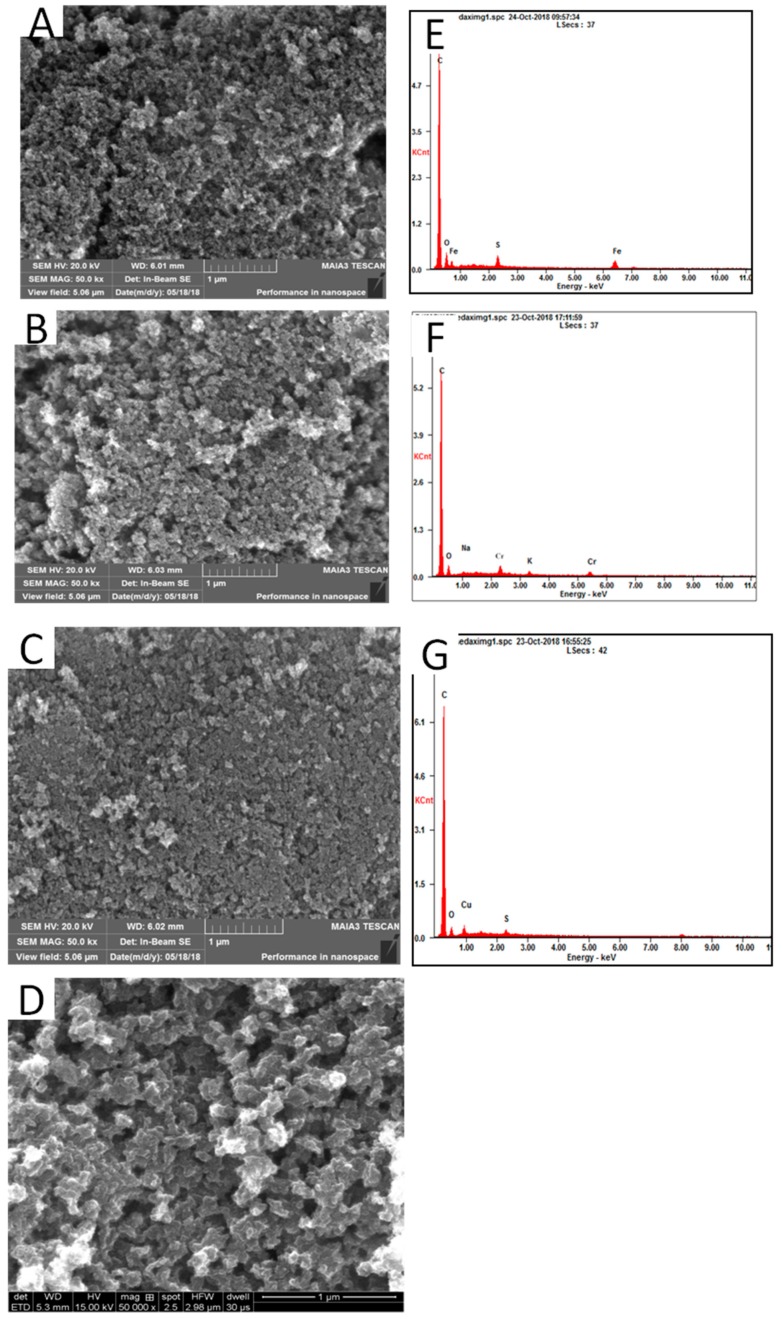
SEM images of CA-X (**A**) CA-Fe (**B**) CA-Cr (**C**) CA-Cu and (**D**) Blank, EDS of CA-X (**E**), CA-Fe (**F**) and CA-Cr (**G**).

**Figure 7:**
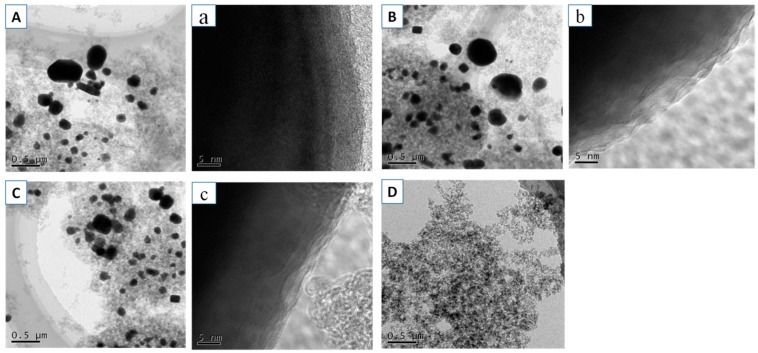
TEM images of (**A**) CA-Fe, (**B**) CA-Cr, (**C**) CA-Cu, and (**D**) Blank, HRTEM images of (**a**) CA-Fe, (**b**) CA-Cr and (**c**) CA-Cu.

Figure 6 presented the SEM images and EDS results of the morphologies of the samples. All samples had a nanostructure. Compared with that of the blank, the structures of CA-Fe, CA-Cr, and CA-Cu were more compact. As seen in Figure 6C, CA-Cu had a more dense porous structure, which was caused by more metal ion adsorption. The EDS was measured to identify the element. According to the Figure 6G, we could confirm the CA-Fe, CA-Cr and CA-Cu had the existence of Fe, Cr, and Cu, respectively. Figure 7 showed the TEM images of the CA-X and blank. Figure 7a–c showed a typical TEM image of the metallic oxide nanosphere, which indicated the appearance of Fe, Cr, and Cu in the Cas [35,36,37].

### 3.4. XRD Patterns of CA-X

**Figure 8:**
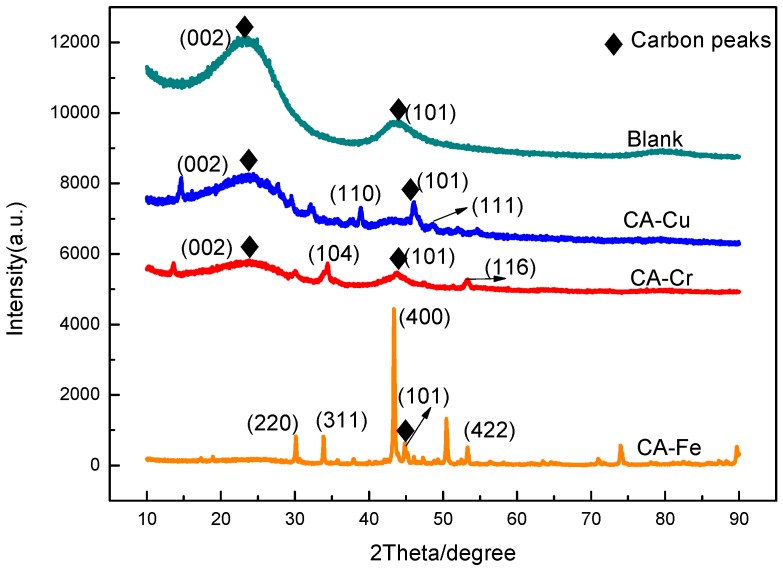
XRD patterns of CA-X.

Figure 8 showed the XRD patterns for the CA-Fe, CA-Cr, CA-Cu, and the blank materials. The CA-Cr, CA-Cu, and blank exhibited typical XRD peaks that could be indexed to (101) and (002), which showed that the CAs were composed of graphite carbon and amorphous carbon. However, CA-Fe did not have the (002) characteristic peak that might be due to the intensity of the iron oxide peaks and an increased average particle size. The loss of the active functional groups could decrease the adsorption capacity. The diffraction peaks located at 2*θ* = 30.1°, 35.8°, 43.1°, and 53.8° could be indexed to the (220), (311), (400), and (422) planes of Fe_3_O_4_, respectively [35]. The curve of CA-Cr could be indexed to (104) and (116), which were characteristic of Cr_2_O_3_ [38,39]. Regarding the sample of CA-Cu, the diffraction peaks at 38.9° and 49.0° matched the respective (110) and (111) planes of CuO. In the above analysis, Fe, Cr, and Cu ions were absorbed into the CAs.

### 3.5. XPS Spectra of CA-X

**Figure 9:**
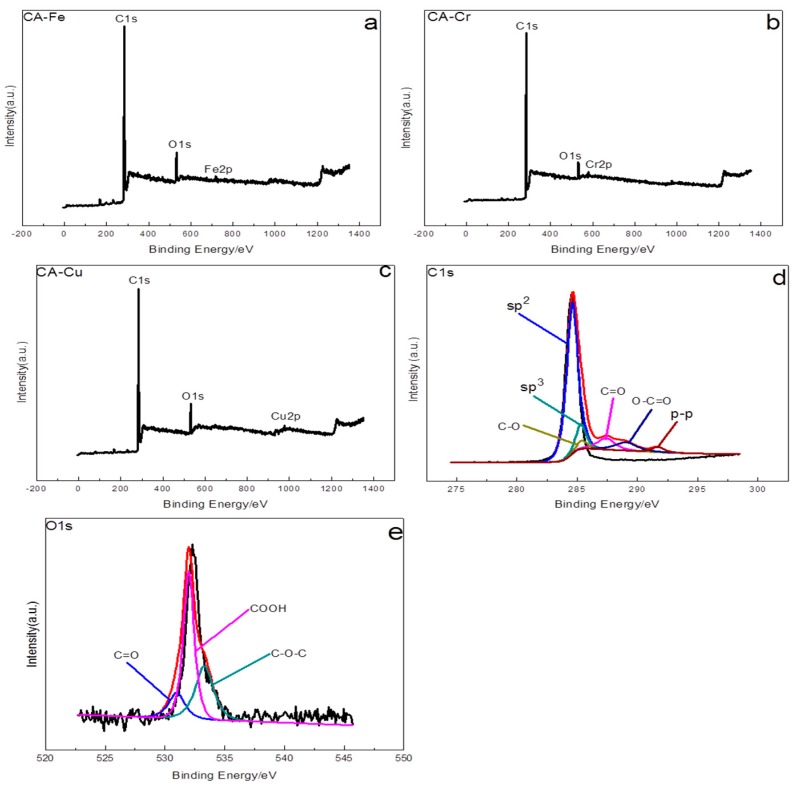
XPS spectra of CA-X, full-scan spectrum (**a**,**b**,**c**), C_1s_ (**d**), and O_1s_ (**e**).

Figure 9a–c showed the high-resolution XPS spectra of CA-Fe, CA-Cr, and CA-Cu, respectively. It was clear that in the CA existed C_1s_ and O_1s_ from Figure 9d,e. The O_1s_ spectra possessed three peaks at 531 eV (C=O), 533.3 eV (C-O-C), and 532 eV (COOH). The C_1s_ spectrum could be fitted into six peaks at 284.6 eV, 285.3 eV, 287.4 eV, 285.4 eV, 289.05 eV, and 291.7 eV corresponding to SP^2^, SP^3^, C=O, C-O, O-C=O and π-π, respectively [40,41]. It was known that these surface functional groups were favorable for metal adsorption because these functional groups can coordinate with metals to form coordinate bonds [42].

### 3.6. Electrochemical Performances of CA-X

**Figure 10:**
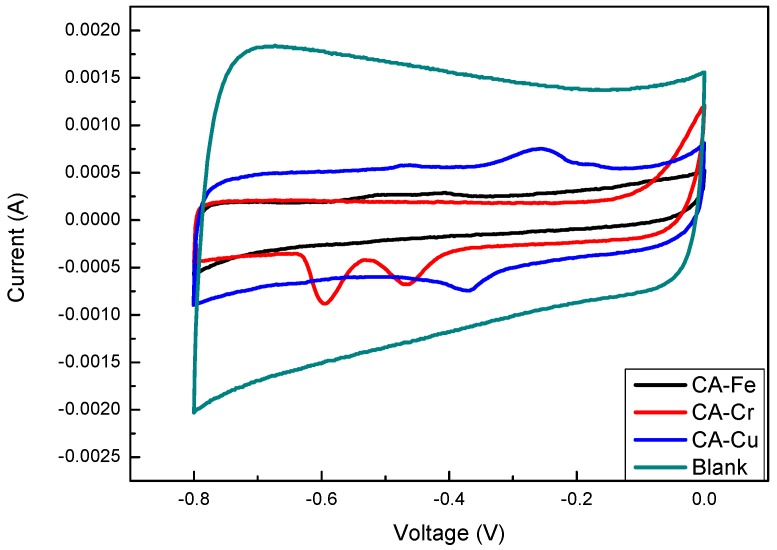
CV curves of different CA-X at a scan rate of 0.5 mV·s^−1.^

The electrochemical properties of CA-Fe, CA-Cr, CA-Cu, and the blank were investigated in a three-electrode system by using 6 M KOH aqueous as the electrolyte. The CV curves acquired at a scan rate of 0.5 mV·s^−1^ were shown in Figure 10. The four kinds of samples showed sharp peaks indicative of the metals. The curve of the blank exhibited a rectangular-like shape, which showed the CAs had good electric double-layer capacitance behavior. In addition, this indicated that the samples had a nanostructure. CA-Fe had a subtle metal peak located at −0.514 and −0.409 V, which indicated that few iron ions were adsorbed and one peak at −0.586 V together with another peak at −0.454 V could be observed in the CV curve of CA-Cr while the peaks at −0.356 and −0.254 V were assigned to CA-Cu, which is shown in Figure 10. All CV curves had two peaks, which implies the good faradaic pseudo capacitance nature [15]. The phenomenon demonstrated that Fe(III), Cr(VI), and Cu(II) have been successfully incorporated into CAs when compared with the blank.

**Figure 11:**
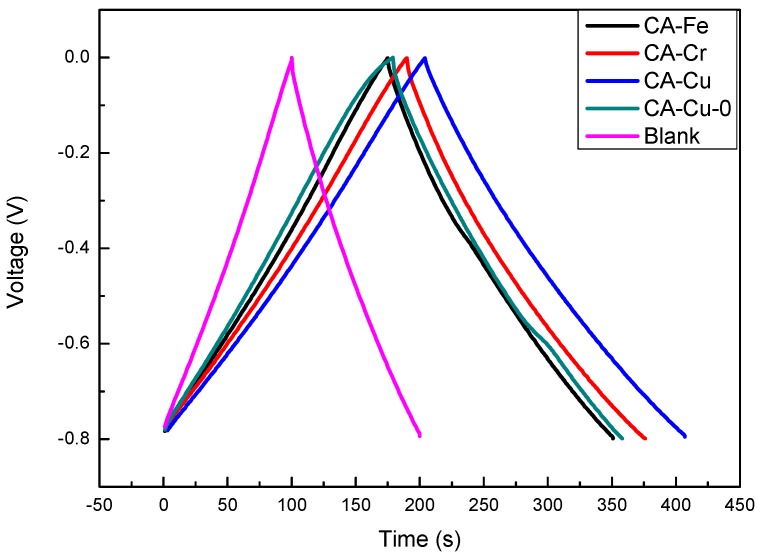
Galvanostatic charge-discharge curves at a current density of 1.0 A·g^−1^.

Figure 11 showed the galvanostatic charge-discharge measurements of CA-Fe, CA-Cr, CA-Cu, CA-Cu-0, and blank at a current density of 1.0 A·g^−1^. The curves were similar to isosceles triangles, which indicated that the charge-discharge had good symmetry and linearly increased or decreased over time. This shape also suggested that this material could serve as a reversible double layer capacitor. In addition, the ion hydration radius (*γ*_H_) was related to the diffusion resistance. *γ*_H_ of Cu(II) was 4.19 Å, *γ*_H_ of Cr(VI) was 4.61 Å, and the *γ*_H_ of Fe(III) was 4.67 Å [43,44]. Clearly, the *γ*_H_ of Cu(II) was the smallest. Smaller *γ*_H_ values indicated lower diffusion resistance, which corresponded to improve charge transfer. The specific capacitance of the CA-Cu was 255 F·g^−1^, the blank was 125 F·g^−1^, and the CA-Fe was 215 F·g^−1^. The specific capacitance data were shown in Table 4. This analysis result demonstrated that CAs not only removed metal ions but also the CAs adsorbing metals could be used as super capacitor materials. From Table 4, we discovered the specific capacitance of CA-Cu was 255 F·g^−1^, which was higher than that of CA-Cu-0. This result showed the copper ions of the CA-Cu-0 could desorb and come into the electrolyte. Table 5 showed the comparison of the super capacitors and similar materials.

**Figure 12:**
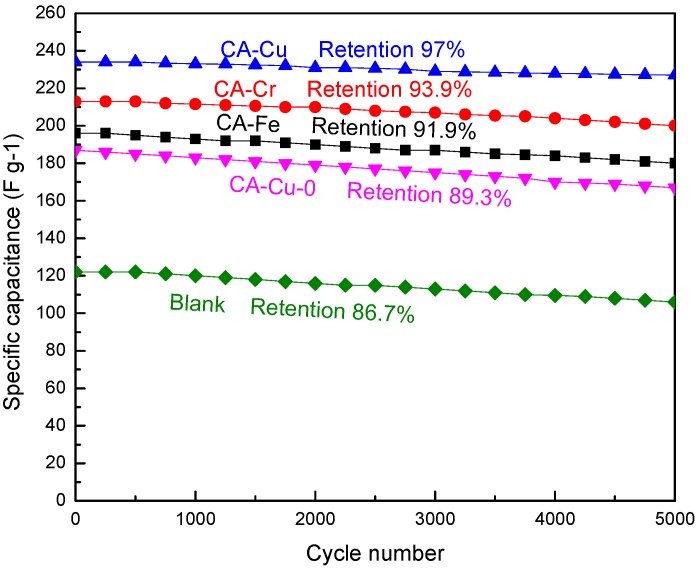
Charge/discharge cycles of CA-X, CA-Cu-0, and blank at the current density of 1.5 A·g^−1.^

Figure 12 showed the durability and stability of CA-X, CA-Cu-0, and the blank. The samples were investigated by galvanostatic charge-discharge at a current density of 1.5 A·g^−1^. It revealed that the specific capacitance of CA-Cu still remained at 227 F·g^−1^ (~97%) after 5000 charge-discharge cycles. Clearly, the CA-Cu displayed good cycling stability. Compared with CA-Cu-0, the CA-Cu had a better stability. At the same time, when compared with CA-Fe and CA-Cr, the better stability features of the CA-Cu might be the compact structures.

**Figure 13:**
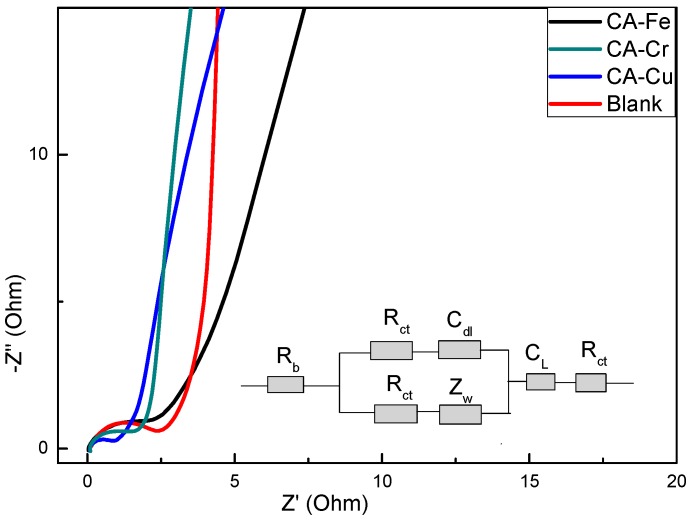
Nyquist plot of CA-X and the blank.

Figure 13 showed the EIS data including the resistance of CA-X and the blank. The frequency of the Nyquist plot ranged from 0.01 to 100 KHz. The plot was divided into two parts: one was the high frequency region, which was a semicircle [49], and the other was the low frequency region, which showed a similarly straight line. The semicircle represented the charge transfer resistance and a smaller radius indicated a smaller charge transfer resistance (R_ct_). The low frequency region reflected the ion diffusion rate, R_b_ was the equivalent circuit resistance, C_dl_ was the stationary phase element, Z_w_ was the Warburg impedance, and C_L_ was the intercalation capacitance. The electrode kinetics characterized the effect of metal adsorption on the pore structure. The EIS method evaluated the apparent chemical diffusion (D_k+_) of the blank and adsorbed metal into the CAs. The K^+^ diffusion coefficient was expressed by the following formula [50].

(6) $$\;Z_{re}=\sigma \omega^{-1/2}\;$$

(7)  Ω=2πf 

(8) $$\;D_{k+}=\frac{R^{2}T^{2}}{2A^{2}n^{4}F^{4}C^{2}\sigma^{2}}\;$$

**Figure 14:**
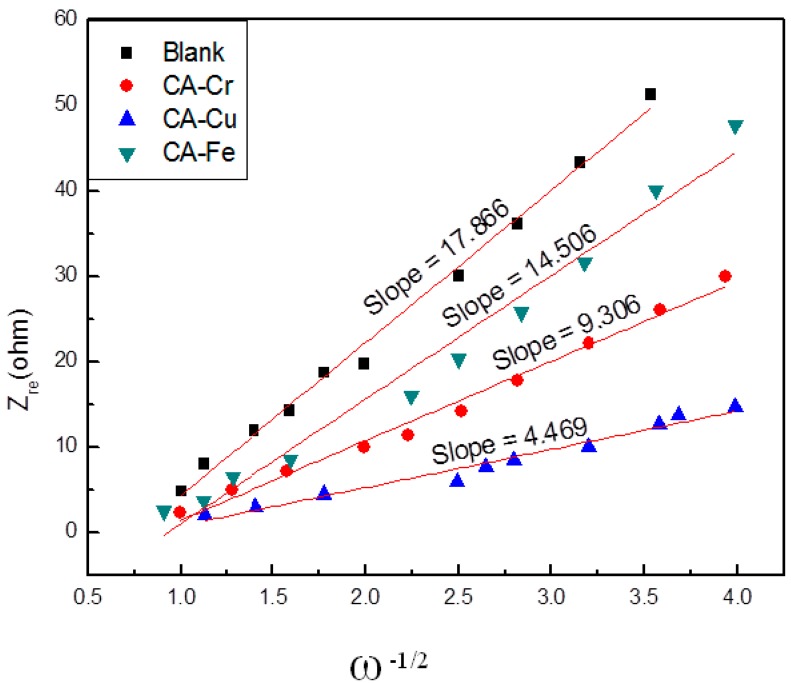
Nyquist graph of Z_re_ plotted against ω^−1/2^ of CA-X and the blank.

In Formula (6), *σ* is the slope of the line and ω is the angular frequency in the low frequency region. In Equation (7), f is the frequency. In addition, A is the specific area of the electrode (0.785 × 10^−4^ m^2^), F is the Faraday constant (9.65 × 10^4^ C·mol^−1^), n represents the number of electrons per molecule involved in the electron transfer, C is the concentration of K^+^ in the KOH solution (6 × 10^3^ mol·m^3^), T is the experimental temperature (298 K), and R is the gas constant (8.314 J·K^−1^·mol^−1^). Figure 14 shows the slope. Combined with Equations (6–8), the K^+^ diffusion coefficients in the CA-Fe, CA-Cr, CA-Cu and blank samples are 0.76 × 10^−15^, 1.8 × 10^−15^, 8.0 × 10^−15^ and 0.5 × 10^−16^cm^2^·s^−1^, respectively. It could be found that the D_k+_ values of CA-X and blank are in the magnitude of 10^−15^ cm^2^·s^−1^ and the diffusion coefficients of K^+^ are increased when the R_ct_ increases. This phenomenon revealed that the kinetics of K^+^ and electro transfer into the electrodes were much faster at low R_ct_. Among these samples, K^+^ diffusion coefficients in the CA-Cu was the biggest. Therefore, the charge transfer resistance of CA-Cu was the smallest.

## 4. Conclusions

In summary, CAs with a higher specific surface area were synthesized through the sol-gel process and the prepared materials exhibited an excellent adsorption capacity. At the same time, the metal ions adsorption on carbon aerogels executed a noticeable improvement for high super capacitor performance. In the adsorption tests, CAs demonstrated a high adsorption capacity for Cu (II), which could reach 424 mg·g^−1^. The dynamics equation confirmed to the pseudo-second-order model and adsorption behavior could be described by the Langmuir adsorption isotherm equation, which indicated the adsorption process was single layer adsorption. The structure of CA-X could be more compact. In addition, metal-adsorbed carbon aerogel materials could be used as super capacitors. According to the results of electrochemical measurement, the electrochemical performance of CAs with adsorbed Cu(II) exhibited the highest specific capacitance, which reached 255 F·g^−1^ at 1.0 A·g^−1^. The excellent adsorption performance indicated that the carbon aerogel was a potential and effective absorbent for metal ions removal and the corresponding carbon aerogels could be used for super capacitors.

## Figures and Tables

**Table 1:** The adsorption capacity of CA-X.

Entry	Adsorption Capacity (mg·g^−1^)
CA-Fe	133
CA-Cr	139
CA-Cu	424

**Table 2:** Comparative study of different adsorbent for the removal of metal ions.

Adsorbents	Metal Ions	Adsorption Capacities (mg·g^−1^)	Reference
Active carbon	Fe	166.7	[28]
Carbon paper@Magnesium silicate	Cu	113.5	[29]
carbon nanotubes	Cu	123.7	[16]
Magnetic b-cyclodextrin/graphene oxide	Cr	120.19	[30]
Carbon bead-supported hollow carbon nanofibers	Cr	51	[31]
Carbon aerogel	Cu/Cr/Fe	424/139/133	This work

**Table 3:** Pore structure of the CA-X.

Entry	S_BET_ (m^2^·g^−1^)	S_micro_ (m^2^·g^−1^)	D_average_ (nm)	V_total_ (cm^3^·g^−1^)
CA-Fe	666	413	8.00	1.33
CA-Cr	627	374	7.50	1.18
CA-Cu	450	274	7.16	0.81
Blank	695	463	8.11	1.52

**Table 4:** The specific capacitance of CA-X and the blank at different current densities.

Entry	Specific Capacitance (F·g^−1^)		
	0.5 A·g^−1^	1.0 A·g^−1^	1.5 A·g^−1^
CA-Fe	239	215	196
CA-Cr	256	238	213
CA-Cu	275	255	234
CA-Cu-0	250	224	186
Blank	129	125	122

**Table 5:** Comparison of the specific capacitance of different carbon materials.

Carbon Species	Metal Species	Capacitance (F·g^−1^)	Current Density (A·g^−1^)	Reference
Carbon fiber	V	104.05	0.5	[15]
Onion-like carbon	Fe	251.2	0.5	[45]
Active carbon aerogels	Mn	152	1.0	[46]
Carbon	Ta	223	1.0	[47]
Graphene	Zr-MOFs	302	0.15	[48]
Carbon aerogel	Cu	255	1.0	This work
